# Complete Chloroplast Genomes of *Vachellia nilotica* and *Senegalia senegal*: Comparative Genomics and Phylogenomic Placement in a New Generic System

**DOI:** 10.1371/journal.pone.0225469

**Published:** 2019-11-25

**Authors:** Sajjad Asaf, Arif Khan, Abdul Latif Khan, Ahmed Al-Harrasi, Ahmed Al-Rawahi

**Affiliations:** 1 Natural and Medical Sciences Research Center, University of Nizwa, Nizwa, Oman; 2 Genomics Group, Faculty of Biosciences and Aquaculture, Nord University, Bodo, Norway; Osmania University, INDIA

## Abstract

*Vachellia* and *Senegalia* are the most important genera in the subfamily Mimosoideae (Fabaceae). Recently, species from both genera were separated from the long-characterized *Acacia* due to their macro-morphological characteristics. However, this morpho-taxonomic differentiation struggles to discriminate some species, for example, *Vachellia nilotica* and *Senegalia senegal*. Therefore, sequencing the chloroplast (cp) genomes of these species and determining their phylogenetic placement via conserved genes may help to validate the taxonomy. Hence, we sequenced the cp genomes of *V*. *nilotica* and *S*. *senegal*, and the results showed that the sizes of the genomes are 165.3 and 162.7 kb, respectively. The cp genomes of both species comprised large single-copy regions (93,849~91,791 bp) and pairs of inverted repeats (IR; 26,093~26,008 bp). The total numbers of genes found in the *V*. *nilotica* and *S*. *senegal* cp genomes were 135 and 132, respectively. Approximately 123:130 repeats and 290:281 simple sequence repeats were found in the *S*. *senegal* and *V*. *nilotica* cp genomes, respectively. Genomic characterization was undertaken by comparing these genomes with those of 17 species belonging to related genera in Fabaceae. A phylogenetic analysis of the whole genome dataset and 56 shared genes was undertaken by generating cladograms with the same topologies and placing both species in a new generic system. These results support the likelihood of identifying segregate genera from *Acacia* with phylogenomic disposition of both *V*. *nilotica* and *S*. *senegal* in the subfamily Mimosoideae. The current study is the first to obtain complete genomic information on both species and may help to elucidate the genome architecture of these species and evaluate the genetic diversity among species.

## Introduction

*Senegalia senegal* (L.) Britton and *Vachellia nilotica* (L.) P.J.H. Hurter & Mabb are the most important species of the genera *Senegalia* and *Vachellia*, which belong to the family Fabaceae [1]. *S*. *senegal* was formerly known as *Acacia senegal* (L.) Wild, and *V*. *nilotica* was known as *Acacia nilotica* [2]. Both species were placed in different genera due to their morphological and taxonomical differences. *S*. *senegal* is a deciduous tree native to arid and semi-desert regions of sub-Saharan Africa but can also be found in other parts of the world, such as the Indian sub-continent and the Arabian peninsula [3]. The genera are well-known for their exudate gum arabic, a non-timber forest product in international trade possessing medicinal, ecological and commercial importance [3]. The gum derived from the tree is used in such industries as food, pharmaceutical and cosmetics [4]. Moreover, this gum is also used in lithographic ink due to its unique emulsification, encapsulation and film-forming properties, adding to the commercial importance of these species [5, 6]. Furthermore, *S*. *senegal* has been noted to increase soil fertility through efficient nutrient fixation, whereas the tree provides shade, fodder, wood fuel [7]. In terms of medicinal uses, gum and tree parts have been known to play bioactive roles in cancer, inflammation, oxidative stress and abdominal complications [7, 8].

In a similar vein, *V*. *nilotica*, a multipurpose legume tree and drought-resistant species, has been well-regarded as a means of rehabilitating dry land ecosystems [9]. This tree increases soil organic carbon, total and available forms of nitrogen and phosphorus under its canopy and can thus be used in soil amelioration [10]. Nitrogenous fertilizers are highly expensive for large-scale afforestation [11]. Utilizing alternative species, such as *V*. *nilotica*, can assist in fixing atmospheric nitrogen to increase soil fertility [5]. The nutrients generated by *V*. *nilotica* trees through biological nitrogen fixation can be exploited within the production system, either simultaneously as an intercropping plant or sequentially, as in rotational fallow systems [9]. *V*. *nilotica* has also been well-documented to possess essential chemical constituents that have been suggested to play roles in fighting cancer, microbial pathogenesis, inflammation, sexually transmitted diseases, oxidative stress, diabetes and mutagenesis[12, 13]. Despite the strong medicinal and local uses of both *V*. *nilotica* and *S*. *senegal*, the taxonomy of these species has not been elucidated. These two species were formerly placed in the genus *Acacia*, despite their major variation from the other species of *Acacia* [14]. The genus Acacia comprised 1350 species distributed in most of the continents, except Antarctica [13]. The 2011 IBC (International Botanical Congress) meeting in Melbourne finally ratified the previous decision, despite the long-standing controversy, paving the way for name changes to *Vachellia* for a smaller and pan-tropical group [15]. This meeting suggested the use of the genera *Senegalia* and *Vachellia* in the classification of *S*. *senegal* and *V*. *nilotica*, respectively. Morphological, biochemical, and palynological data are highly important for the classification of plants into their respective genera [16]. However, emphasis has been placed exegetically to further understand and create more genomic datasets to elucidate these difficult-to-classify and important species [17].

In this regard, chloroplast, the most important organelle in plant cells, plays an important role in photosynthesis, carbon fixation, fatty and amino acid synthesis [18, 19] and has been a focus of attention in recent decades to understand taxonomy, evolution and biological processes. Ideally, a chloroplast (cp) genome of angiosperms exhibits a quadripartite structure size ranging from 110 kb -160 kb. The quadripartite structure is usually composed of a large single copy (LSC) region, a small single copy region (SSC) region and a pair of inverted repeats (IR), which are mirror images of each other [19]. Angiosperm cp genomes generally contain 80 protein-coding genes, 4 ribosomal RNA (rRNA) genes, and 30 transfer RNA (tRNA) genes [20]. The majority of cp genomes exhibit highly conserved structures, some reveal structural variations, IR loss, and gene loss as a result of adaptation to their environments [21, 22]. Next-generation technologies have allowed the rapid sequencing of many cp genomes in recent years [23]. These abundant cp genomes have facilitated the verification of evolutionary relationships and allowed detailed phylogenetic classifications to be conducted at the group, family, and even generic level in plants [24, 25]. Furthermore, cp genomes can be used for species identification through DNA barcoding and molecular markers that enable morphologically similar species to be distinguished [26]. Despite the highly economic, biological, ecological and social importance of these genera, very little information is available on the comparative chloroplast genomes of *Senegalia* and *Vachellia*. It is difficult to demarcate monophyletic lineages within these genera, despite morphological differences, and they face classification issues [27, 28]. In this study, we sequenced the chloroplast genomes of *V*. *nilotica* and *S*. *senegal*, and complete phylogenomic analysis was performed to validate their placement in the genera *Vachellia* and *Senegalia*, respectively. Our study provides sequence resources for future studies of population diversity and taxonomy.

## Materials and methods

### Chloroplast DNA extraction and sequencing

Young and immature green fresh photosynthetic leaves of *V*. *nilotica* and *S*. *senegal* were ground to fine powder in liquid nitrogen, and the contamination-free chloroplast DNA was isolated according to the modified protocol of Shi et al., [29]. The Ion Torrent sequencing platform was used for sequencing intact chloroplast DNA using the Ion torrent S5 sequencer with the Ion Torrent server (Life Technologies, USA). Genomic libraries were prepared according to the manufacturer’s instructions (Life Technologies, USA). The total chloroplast DNA of each sample was sheared enzymatically into approximately 400-bp fragments using the Ion Shear Plus Reagents kit, and libraries were prepared using the Ion Xpress Plus gDNA Fragment Library kit. Prepared libraries were quantified and qualified on a Qubit 3.0 fluorimeter and an Agilent 2100 Bioanalyzer system. Library preparations were followed by template amplification (Ion one touch 2 instrument, Life Technologies, USA), and enrichment of the amplified template was performed (Ion OneTouch™ ES enrichment system, Life Technologies, USA) using Ion 520 and 530 OT2 reagents. The prepared libraries were loaded onto the Ion S5 sequencing chip, and sequencing was performed according to the Ion torrent S5 protocol (Life Technologies USA).

### Genome assembly

The sequencing of *V*. *nilotica* and *S*. *senegal* resulted in 185,114 and 137,673 reads, respectively. The obtained reads of both *Vachellia* and *Senegelia* species were mapped to the selected reference genome of *Vachellia flava* and *Senegalia laeta* using Bowtie ((v.2.2.3) [30] in Geneious Pro (v.10.2.3) [31] software. The mean coverage of the reads for *V*. *nilotica* and *S*. *senegal* were 134X and 168X, respectively. The IR (inverted repeat) junction regions were selected from the reference genomes to adjust the sequence length, and the iteration method was used with MITObim (v.1.8) [32].

### Genome annotation

Chloroplast genomes of the sequenced species were annotated by using Dual Organellar Genome Annotator (DOGMA)[33], and the number and position of ribosomal RNAs, transfer RNAs and coding genes present in chloroplast genomes were identified and analyzed using BLASTX and BLASTN, and tRNAscan-SE version 1.21 [34] software was used to annotate tRNA genes. Additionally, for manual adjustment, Geneious (v11.0) and tRNAscan-SE [34] were used to compare the genome with previously reported reference genomes. Correspondingly, the start and stop codons and intron boundaries were also manually adjusted compared with the pre-published reference cp genome. In addition, the structural features of the cp genomes of both *V*. *nilotica* and *S*. *senegal* species were illustrated using OGDRAW [35]. Correspondingly, the MEGA6 software [36] was used to determine the relative synonymous codon usage and deviations in synonymous codon usage by avoiding the influence of amino acid composition. The divergence of *V*. *nilotica* and *S*. *senegal* species taxa genomes from those of other related species (Fig 5) was determined using mVISTA [37] in Shuffle-LAGAN mode and using *V*. *nilotica* and *S*. *senegal* as reference genomes.

### Repeat identification

REPuter software [38] was used for the identification of palindromic, forward and tandem repeats present in the genome. The criterion was a minimum >15 base pairs with a sequence identity of 90%. Furthermore, SSRs were determined using Phobos version 3.3.12 [39] with the search parameters set for mononucleotide repeats ≥ 10 repeat units, dinucleotide repeats ≥ 8 repeat units, tri- and tetranucleotide repeats ≥ 4 repeat units, and pentanucleotide and hexanucleotide repeats ≥ 3 repeat units. Tandem Repeats Finder version 4.07 b [40] with default settings was used to determine tandem repeats.

### Sequence divergence and phylogenetic analysis

The average pairwise sequence divergence of the complete cp genomes of *Vachellia* and *Senegalia* species with related species was determined. Comparative sequence analysis after comparing gene order and performing multiple sequence alignment was used to identify missing and ambiguous gene annotations. MAFFT version 7.222 [41], with default parameters was used for the alignment of complete genomes, and pairwise sequence divergence was calculated by selected Kimura’s two-parameter (K2P) model [42]. To resolve the phylogenetic position of *V*. *nilotica* and *S*. *senegal* within the family Fabaceae, cp genomes were downloaded from the NCBI database. Alignment of the complete cp genomes was constructed on the basis of conserved gene order and structure of the cp genome. Four methods were used to infer the phylogenetic trees, including maximum parsimony (MP) implemented with PAUP 4.0100, neighbour-joining (NJ) and maximum likelihood (ML) with MEGA 6[36] and Bayesian inference (BI) with MrBayes 3.1.299 [43] using setting derived from Asaf et al [44] and Wu et al [45]. ML analysis parameters were adjusted with a BIONJ tree with 1000 bootstrap replicates using the Kimura 2-parameter model with gamma-distributed rate heterogeneity and invariant sites. A heuristic search for MP analysis was run with 1000 random addition sequence replicates with the tree-bisection-reconnection (TBR) branch-swapping tree search criterion. The best substitution model GTR + G model was used according to the Akaike information criterion (AIC) by jModelTest version 2102 for Bayesian posterior probabilities (PP) in the BI analyses. The Markov Chain Monto Carlo (MCMC) was run with 4 incrementally heated chains for 1,000,000 generations, starting from random trees and sampling 1 out of every 100 generations. The first 25% of trees were discarded as burn-in to estimate the value of posterior probabilities. In another phylogenetic study, 65 shared genes from the cp genomes of the 102 Fabaceae members downloaded from NCBI were aligned in MAFFT version 7.222 [38]. The above four phylogenetic-inference methods were used to infer trees from these 65 concatenated genes using the same settings described above. The assembled and annotated complete chloroplast genome was submitted to NCBI under the accession numbers MK645904 (*V*. *nilotica*) and MK645903 (*S*. *senegal*).

## Results

### General features of *V*. *nilotica* and *S*. *senegal* chloroplast genomes

**Fig 1:**
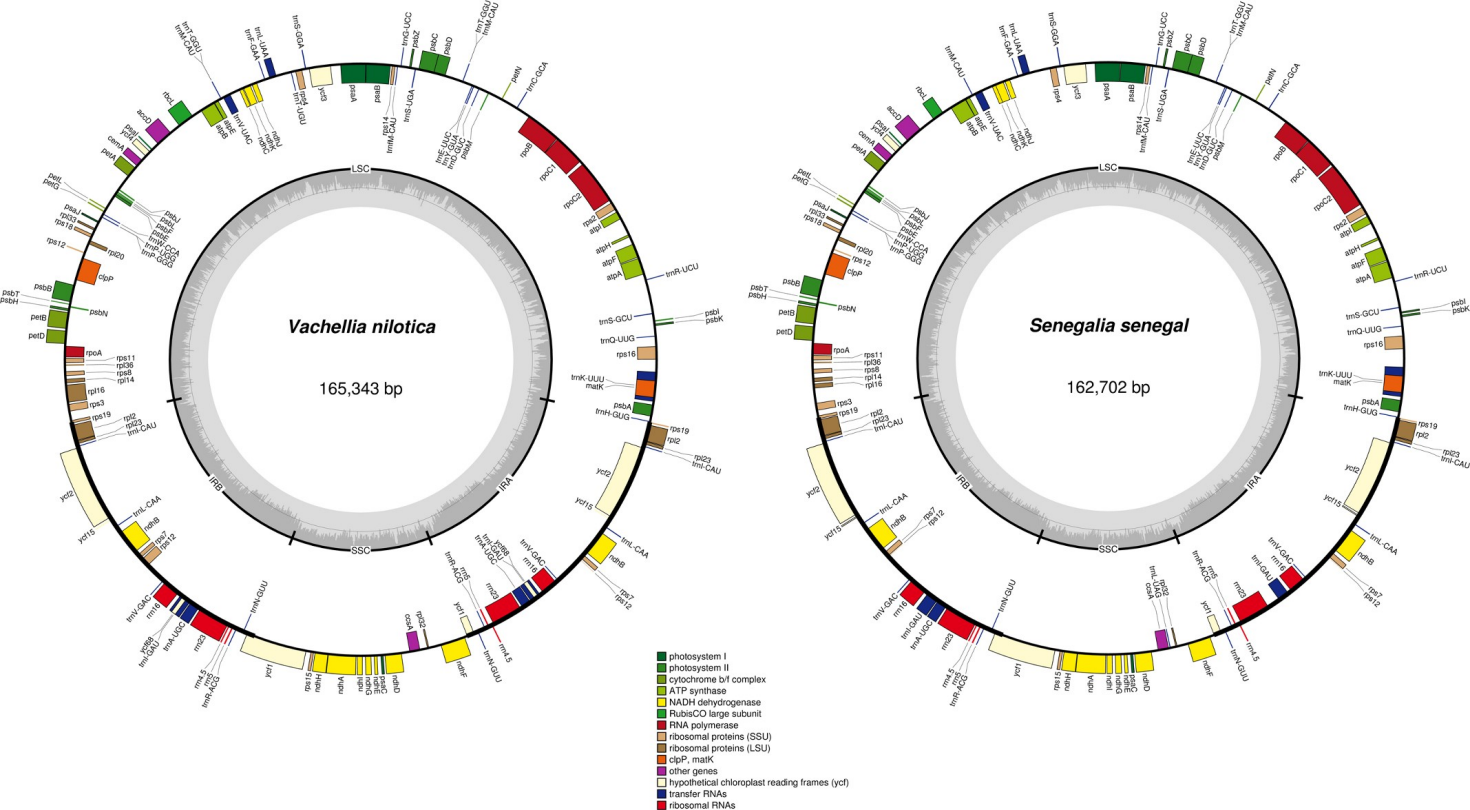
Genome map of the *Vachellia nilotica* and *Senegalia senegal* chloroplast genome. Thick lines indicate the extent of the inverted repeat regions (IRa and IRb), which separate the genome into small (SSC) and large (LSC) single copy regions. Genes drawn inside the circle are transcribed clockwise, and those outside are transcribed counterclockwise. Genes belonging to different functional groups are color-coded. The dark grey in the inner circle corresponds to the GC content, and the light grey corresponds to the AT content.

The complete chloroplast genomes of *V*. *nilotica* and *S*. *Senegal* exhibited typical sizes of 165,343 bp and 162,702 bp, respectively. These genomes showed a typical quadripartite structure with a large single copy region (LSC) and a small single copy region (SSC) and a pair of inverted repeats (Fig 1). The completely sequenced genomes of *V*. *nilotica* and *S*. *Senegal* were compared with seventeen other chloroplast genomes, where the results showed that the sizes of compared genomes ranged from 178,887 bp (*Pithecellobium flexicaula*) to 159,389 bp (*Adenanthera micrsperma*). The overall GC content in *V*. *nilotica* was found (35.4%) to be less than that in *S*. *senegal* (35.7%). The LSC regions were 39,849 bp and 91,791 bp, while the SSC regions were 19,308 bp and 18,895 bp, respectively, in *V*. *nilotica* and *S*. *senegal*. The IR region in the two cp genomes was found to be similar in *V*. *nilotica* (26,093) and *S*. *senegal* (26,008). The number of rRNAs (04) in all the sequenced and compared genomes was the same, while the numbers of tRNAs in the genomes were 37 and 38 in *S*. *senegal* and *V*. *nilotica*, respectively (Table 1).

**Table 1:** Composition of *Vachellia nilotica* and *Senegalia senegal* cp genomes with related species.

	Size (bp)	Overall GC contents	LSC size in bp	SSC size in bp	IR size in bp	Protein coding regions size in bp	tRNA size in bp	rRNA size in bp	Number of genes	Number of protein coding genes	Number of rRNA	Number of tRNA	Genes with introns
***V*. *nilotica***	165,343	35.4	93849	19308	26093	79520	2847	9052	135	89	8	38	22
***V*. *seyal***	165,383	35.3	93901	19347	26068	78009	2828	9052	127	82	8	37	21
***V*. *flava***	165,829	35.3	94220	19474	26068	78033	2828	9052	127	82	8	37	21
***S*. *Senegal***	162702	35.7	91791	18895	26008	75571	2794	9052	132	87	8	37	22
***S*. *Laeta***	162754	35.8	91750	18911	26047	77997	2829	9052	127	82	8	37	20
***S*. *saman***	176717	35.3	92036	5053	39814	89202	2793	9052	138	92	8	37	21
***p*. *flexicaula***	178887	35.1	91076	4805	41503	89784	2793	9078	139	94	8	37	23
***P*. *communis***	162,552	35.9	91517	18941	26047	77949	2793	9052	130	83	8	37	23
***P*. *javanica***	161,681	35.9	91093	18574	26007	78075	2794	9052	130	83	8	37	23
***P*. *pruinosum***	176,692	35.3	92320	5036	39668	89271	2793	9052	138	92	8	37	24
***L*. *trichandra***	164692	35.6	93690	18890	26056	78759	2815	9049	129	84	8	37	22
***I*. *leiocalycina***	175489	35.5	90987	4948	39777	89244	2820	9056	137	92	8	37	24
***F*. *albida***	175646	35.3	91045	4761	39920	88638	2793	9068	138	90	8	37	23
***D*. *cinerea***	161240	35.9	90430	18526	26142	77958	2793	9068	130	83	8	37	23
***A*. *lucyi***	176870	35.2	92319	4573	39989	89133	2793	9052	138	92	8	37	24
***A*. *odoratissima***	174861	35.6	90169	4928	39882	89130	2793	9066	138	92	8	37	24
***A*. *microsperma***	159389	36.5	88577	18756	26028	78030	2793	9052	130	83	8	37	23
***A*. *ligulata***	174233	35.4	92770	4986	38225	88107	2802	9062	134	89	8	37	22
***A*. *daelbata***	174217	35.4	92753	4956	38254	88551	2793	9060	137	91	8	37	22

### Important genes and base composition in sequenced cp genome

Furthermore, the gene content, gene size and gene order of *V*. *nilotica* and *S*. *senegal* were largely similar, comprising 135 and 132 genes, respectively. Among all the compared genomes, *P*. *flexicaula* possessed the highest number of genes (139), and *S*. *laeta* showed the lowest number of genes (127). The numbers of protein coding genes (PCGs) were 89 and 87 in *V*. *nilotica* and *S*. *senegalia*, respectively. However, these numbers were found to be highest (94) in the *P*. *flexicaula* cp genome. The PCGs in the chloroplast genome include some important genes responsible for photosynthesis, i.e., Photosystem I (*psaA*, *B*, *C*, *I*, *J*) and Photosystem II (*psbA*, *B*, *C*, *D*, *E*, *F*, *H*, *I*, *J*, *K*, *L*, *M*, *N*, *T*, *Z*). The genes responsible for tRNA, rRNA, large subunit and small subunit of ribosomal proteins were also present in the chloroplast genome. Other important genes annotated in the chloroplast genome were *matK*, *clpP*, *cemA*, *accD*, *ccsA*, *ycf1*, *3*, *4*, *15*, which were also present in the chloroplast genome (Table 2). Approximately 22 intron-containing genes were observed in both sequenced genomes (Table 1).

**Table 2:** Genes in the sequenced *V*. *nilotica* and *S*. *senegal* species chloroplast genomes.

Category	Group of genes	Name of genes
**Self-replication**	Large subunit of ribosomal proteins	*rpl2*, *14*, *16*, *20*, *22*, *23*, *32*, *33*, *36*
	Small subunit of ribosomal proteins	*rps2*, *3*, *4*, *7*, *8*, *11*, *12*, *14*,*15*, *16*, *18*, *19*
	DNA dependent RNA polymerase	*rpoA*, *B*, *C1*, *C2*
	rRNA genes	*rrn 4*.*5*, *rrn 5*, *rrn 16*, *rrn23*
	tRNA genes	*trnC-GCA*, *trnD-GUC*, *trnfM-CAU*, *trnG-UCC*, *trnH-GUG*, *trnI-CAU*, *trnI-GAU*, *trnK-UUU*, *trnL-CAA*, *trnL-UAA*, *trnL-UAG*, *trnM-CAU*, *trnN-GUU*, *trnP-GGG*, *trnP-UGG*, *trnQ-UUG*, *trnR-ACG*, *trnR-UCU*, *trnS-GCU*, *trnS-GGA*, *trnS-UGA*, *trnT-GGU*, *trnT-UGU*, *trnV-UAC*, *trnW-CCA*, *trnY-GUA*
**Photosynthesis**	Photosystem I	*psaA*, *B*, *C*, *I*, *J*,
	Photosystem II	*psbA*, *B*, *C*, *D*, *E*, *F*, *H*, *I*, *J*, *K*, *L*, *M*, *N*, *T*, *Z*
	Cytochrome b6/f complex	*petA*, *B*, *D*, *G*, *L*, *N*
	ATP synthase	*atpA*, *B*, *E*, *F*, *H*, *I*
	Rubisco	*rbcL*
**Other genes**	Maturase	*matK*
	Protease	*clpP*
	Envelop membrane protein	*cemA*
	Subunit Acetyl- CoA-Carboxylate	*accD*
	c-type cytochrome synthesis gene	*ccsA*
**Unknown**	Conserved open reading frames	*ycf1*, *3*,*4*, *15*

In the complete genome, the composition of (T) is higher than other base nucleotides present in the genome, which is 32.9% and 32.7% in *V*. *nilotica* and *S*. *senegal*, respectively. Adenine (A), which comprises the first position in both *V*. *nilotica* and *S*. *senegal*, accounts for 34.47 and 30.8, respectively. The (T/U) base at the 2^nd^ position was found to be higher than the other genomes, accounting for 33.59 and 32.2 in *V*. *nilotica* and *S*. *senegal*, respectively. Similarly, the (T/U) base was also found to be abundant at the 3^rd^ position (Table 3).

**Table 3:** Base composition of the *Vachellia nilotica and Senegalia senegal* chloroplast genome.

	T/U		C		A		G		Length (bp)	
	V.N	S.S	V.N	S.S	V.N	S.S	V.N	S.S	V.N	S.S
**Genome**	32.9	32.7	17.9	18	31.7	31.6	17.5	17.7	165343	162702
**LSC**	34.6	34.3	16.7	16.9	32.9	32.7	15.8	16	93849	91791
**SSC**	35.8	35.8	14.2	14.4	34.5	34.2	15.5	15.5	193.8	18895
**IR**	28.4	28.3	22.1	22.1	28.9	29.1	20.6	20.6	26093	26008
**tRNA**	25.3	24.9	23.3	23.4	22.1	22.2	29.3	29.5	2847	2794
**rRNA**	19	18.9	23.7	23.7	25.6	25.7	31.7	31.7	9052	9052
**Protein Coding genes**	31.9	32.1	17.4	17.3	30.6	30.7	20.1	19.9	79520	75571
**1st position**	23.36	25.2	17.43	18.20	34.47	30.8	25.57	25.16	**26506**	25189
**2nd position**	33.59	32.2	19.09	19.78	29.26	29.62	19.44	17.49	**26506**	25189
**3rd position**	40.12	38.7	15.24	13.8	27.82	31.6	15.15	14.4	**26506**	25189

V.N = *Vachellia nilotica*, S. S = *Senegalia Senegal*.

### Comparison of sequenced genomes with other genomes

Comparison of the currently two sequenced and seventeen other genomes from the database (NCBI) revealed that the *P*. *flexicaula* (178,887 bp) cp genome was the largest, and that of *A*. *microsperma* (159,389) was the smallest (Table 1). *V*. *nilotica* contains the highest number of tRNAs (38) among all the compared genomes. The highest number of genes was found in the *P*. *flexicaula* (139), and the lowest number was found in 127 genes and was similar in the *V*. *seyal*, *V*. *flava* and *S*. *laeta* chloroplast genomes. The highest number of PCGs (protein coding genes) was observed in *P*. *flexicaula* (94 genes), and the lowest number (82) was found to be similar in *V*. *seyal*, *V*. *flava* and *S*. *laeta*. The number of rRNAs was similar in all of the compared and sequenced chloroplast genomes, while the number of tRNA- and intron-containing genes varied in all of the chloroplast genomes (Table 1). The largest LSC region was found in *V*. *flava* (94,220 bp), and the smallest LSC was observed in *A*. *microsperma* (88,577 bp), which is also the smallest genome. The largest SSC region was found in *V*. *flava* (19,474 bp), while the smallest was found in *A*. *lucyi* (4,573 bp).

### Comparative sequence divergent regions in genome

The complete chloroplast genomes of *V*. *nilotica* and *S*. *senegal* were compared with seventeen species for sequence divergent regions from the NCBI database using mVISTA [37]. The comparative analyses of the chloroplast genome showed a high level of similarity. Overall, the comparison of these chloroplast genomes observed similarity in coding regions, while non-coding regions had more variation, which is almost two times that of coding regions (S1 Fig). The *V*. *nilotica* chloroplast genome was used as a reference genome. The comparative analyses of *V*. *nilotica* with related species revealed high sequence similarity with no obvious difference from *V*. *flava*. The most variable coding regions found in these genomes are *tran K*, *rps16*, *rpoC1*, *petB*, *petD*, *ycf2*, *rrn23*, and *ndhA*. In particular, the *ycf1* gene displayed more variation among all variable regions (S1 Fig).

### Analysis of repetitive sequences in genomes

**Fig 2:**
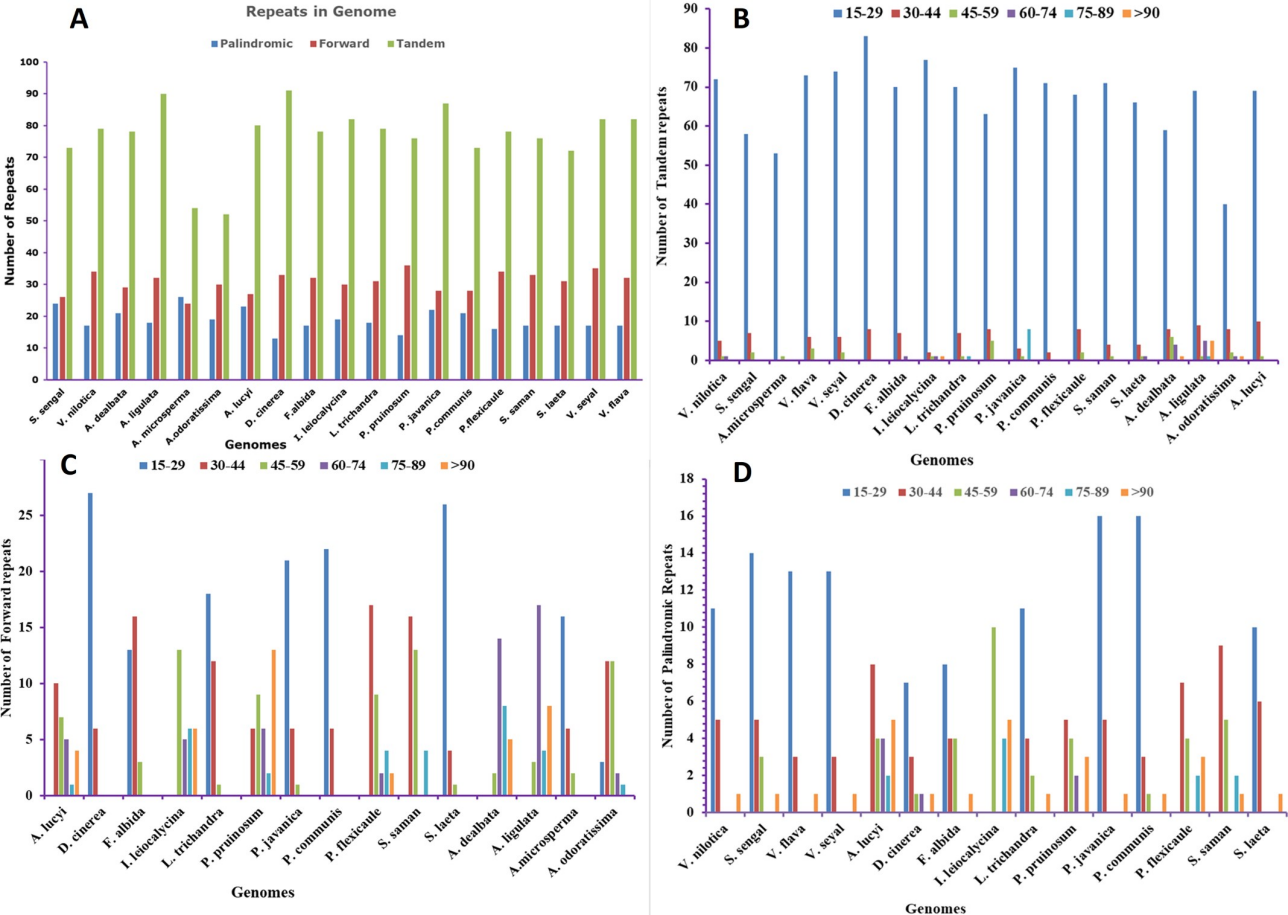
Analysis of repeated sequences in *V*. *nilotica* and *S*. *senegal*. **(A)** Totals of three repeat types, **(B)** Frequency of palindromic repeats by length, **(C)** Frequency of forward repeats by length and (**D)** Frequency of tandem repeats by length.

Repeat analysis of the sequenced cp genomes showed that there were 123 repeats in the *S*. *senegal* cp genome, which comprised 24 palindromic repeats, 26 forward repeats and 73 tandem repeats. Similarly, in *V*. *nilotica*, 130 repeats were present, containing 17 palindromic, 34 forward and 79 tandem repeats (Fig 2). In *V*. *nilotica*, the highest number of repeats was observed, and the sizes ranged from 15–29 in all palindromic, forward and tandem repeats containing 11, 20 and 72 repeats, respectively. A similar trend was observed in *S*. *senegal* containing 15–29 repeat sizes with 18, 23 and 70 repeats, respectively. Analysis of total repeats showed that *V*. *nilotica* had similarity with *Archidendron lucyi* in repeat number, with each containing 130 repeats. Similarly, *V*. *flava* and *V*. *seyal* also had 131 and 134 repeats, which showed similarity in repeat number to *V*. *nilotica*. The other species that was similar to *S*. *senegal* regarding repeats was *S*. *laeta*, containing 120 repeats, suggesting that *S*. *senegal* shows similarity in terms of repeats. Overall, in the compared genomes, *Acacia ligulata* comprised the highest number of repeats (140), and *Albizia odoratissima* contained the lowest number of repeats (101) (Fig 2).

### SSRs in the genomes

**Fig 3:**
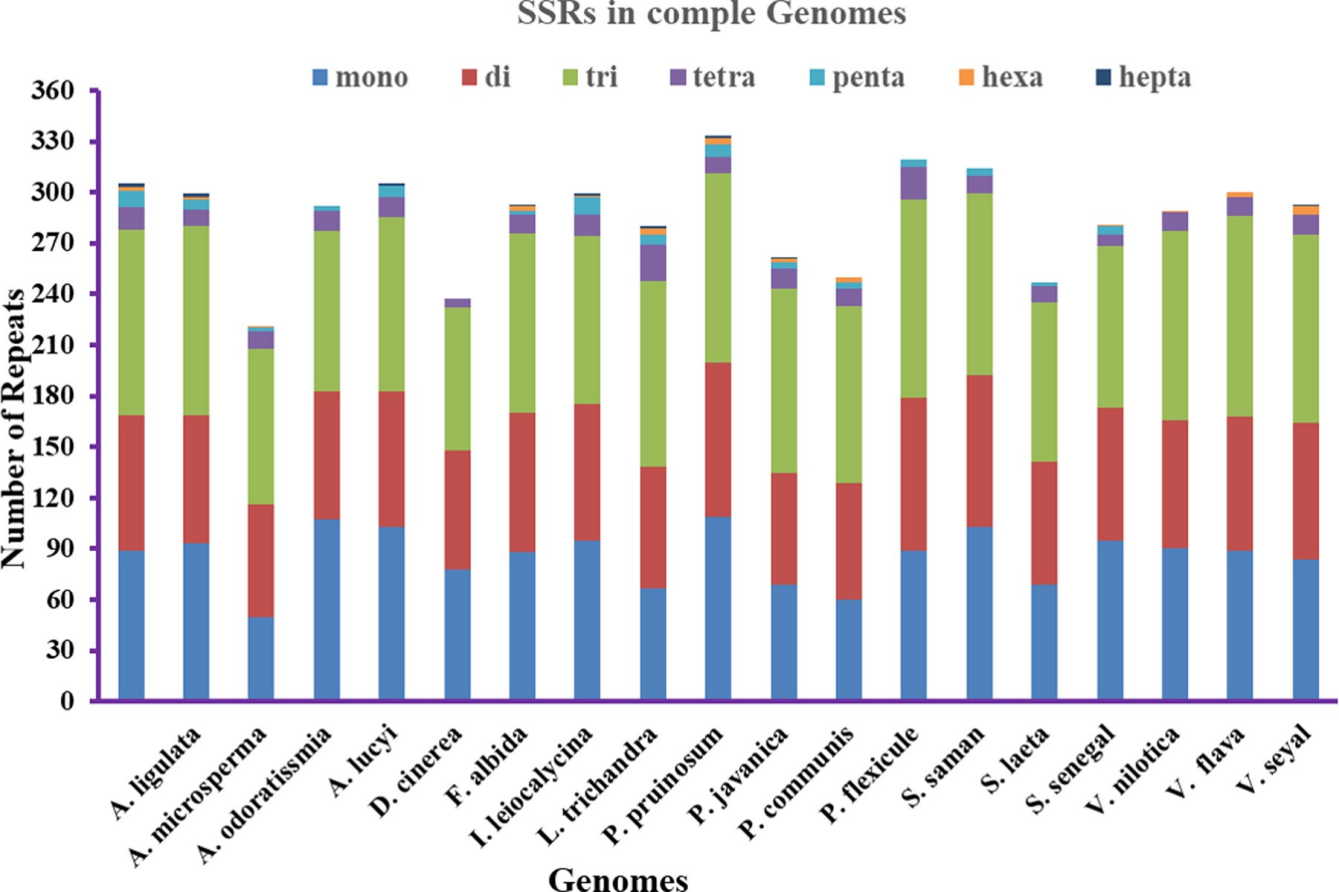
Analysis of simple sequence repeat (SSR) in *V*. *nilotica* and *S*. *senegal* genomes with related species cp genomes. Number of different SSR types detected in these genomes.

**Fig 4:**
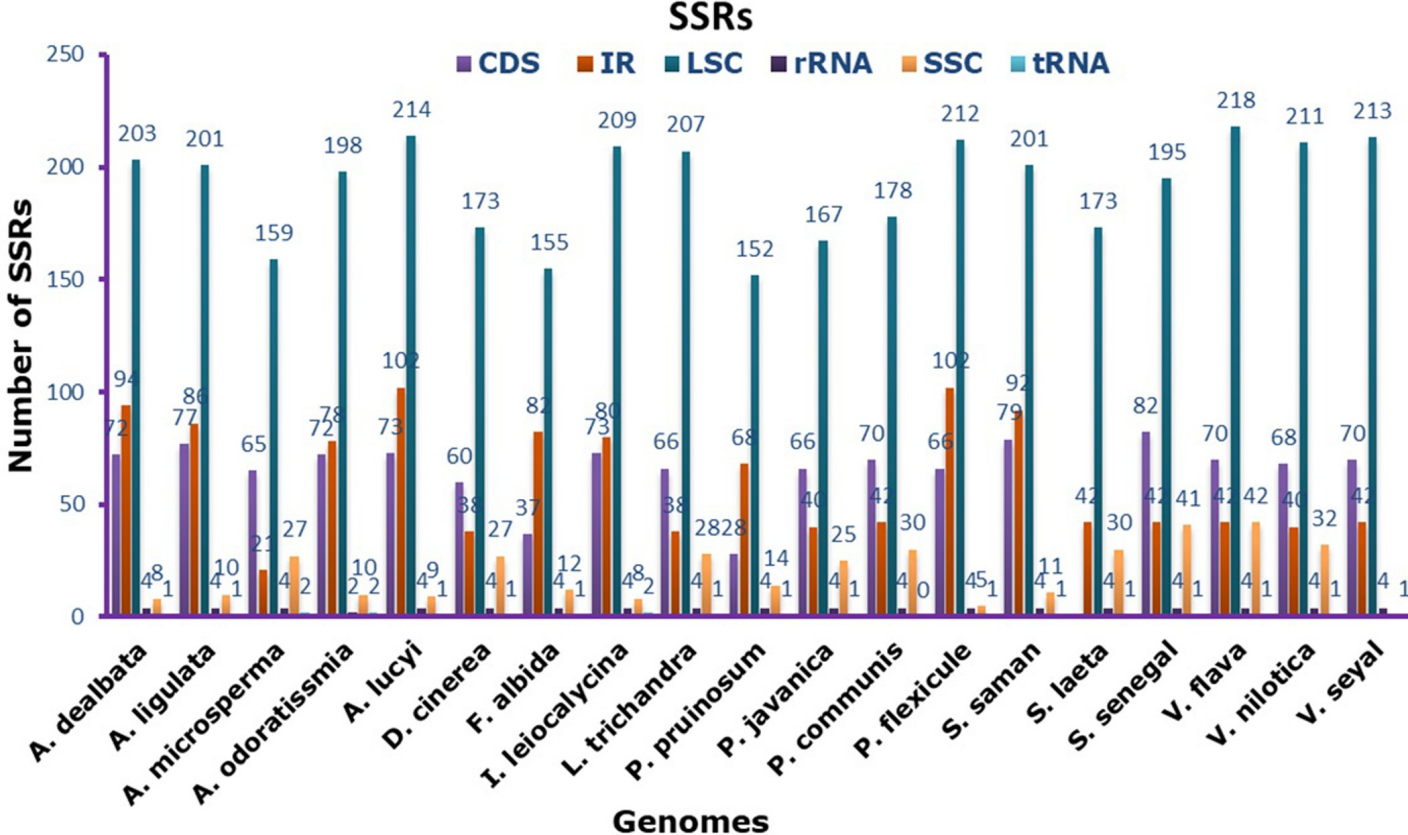
Analysis of simple sequence repeat (SSR) in the *V*. *nilotica* and *S*. *senegal* genomes. Frequency of identified SSRs in the Small Single-Copy (SSC), Large Simple-Copy (LSC), Inverted Repeat (IR), transfer RNA (tRNA), ribosomal RNA (rRNA), and coding sequence (CDS) regions.

The SSRs (1–7) present in the *V*. *nilotica* genome were analyzed, and a total of 290 and 281 SSRs were present in *V*. *nilotica* and *S*. *senegal*, respectively. In *V*. *nilotica*, the most numerous SSRs were trinucleotide repeats (111) followed by mononucleotide (90) and dinucleotide (76) SSRs (Fig 3). The highest number of SSR nucleotides present in the *V*. *nilotica* genome was an octanucleotide (1). Similarly, in *S*. *senegal*, the total number of SSRs found was 281, where the highest number of nucleotides were trinucleotide (95) followed by mononucleotide (94) and dinucleotide repeats (78). Furthermore, *V*. *nilotica* contains the least number of SSRs when compared to other *Vachellia* species, i.e., *V*. *flava* and *V*. *seyal* with 302 and 295 SSR repeats, respectively. *S*. *senegal* had the highest number of SSRs compared to *S*. *laeta*. The number of SSRs was abundant in coding regions of all the sequenced and compared cp genomes (Fig 4). *V*. *nilotica* and *S*. *senegal* had 211 and 195 SSRs in the coding regions, respectively. Furthermore, *V*. *flava* contained the highest number (218) of SSRs in the coding region among all the compared genomes (Fig 4).

### Contraction and expansion of IR regions

Comprehensive comparative analysis of the junction region was performed among the 19 species for the contraction and expansion in J_Lb_ (LSC-IRb), J_SB_ (IRb-SSC), J_SA_ (SSC-IRa), and J_LA_ (IRa-LSC) and also for the position of genes present on these junctions. The largest inverted repeat region was found in the largest chloroplast genome of *P*. *flexicaula*, which was 41,503 bp in size, and the smallest IR region was found in the *P*. *javanica* (26,007 bp) chloroplast genome.

**Fig 5:**
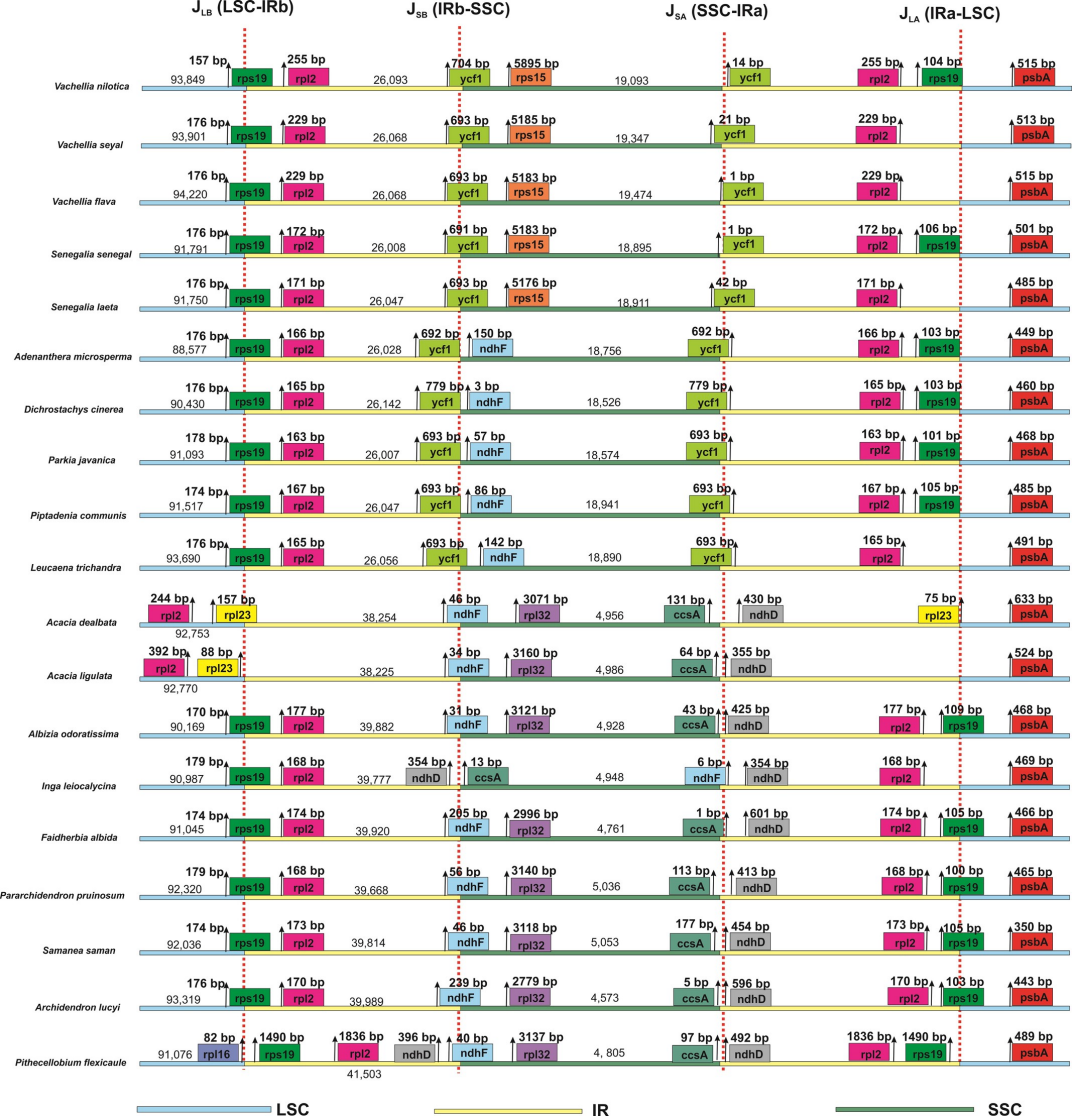
Comparison of border distance between adjacent genes and junctions of LSC, SSC and two IR regions among the chloroplast genomes of *V*. *nilotica* and *S*. *senegal* with related species. Boxes above or below the main line indicate the adjacent border genes. The figure is not to scale with respect to sequence length and only shows relative changes at or near the IR/SC borders.

Although genomic structure and gene composition are highly conserved among these genomes, there are some differences in the IR region. Comparison of the J_SB_ junction of *Vachellia* species (*V*. *nilotica*, *V*. *seyal*, *V*. *flava*) and *Senegalia* species (*S*. *senegal*, *S*. *laeta*) revealed small differences, and the genes at the junction regions are also conserved. In the junction regions of all the compared genomes, the *ycf1* gene is conserved and present at the same position (Fig 5). In the J_SB_ junction in *Vachellia* and *Senegalia* species, the *ycf 1* gene is present, while in the remaining species, it is located in the IRb region. Furthermore, at the J_LB_ junction in all the genomes, the *rpl2* gene is located in the IRb region, except for *A*. *ligulata* and *A*. *dealbata*, in which the *rpl2* gene is present in the LSC region. Moreover, at the J_SB_ junction, the *rps15* gene was found in the SSC region of all *Vachellia* and *Senegalia* species, while other compared genomes were absent. In addition, *rpl23* was only at the J_LB_ junction in *Accacia dealbata*. Similarly, in *S*. *senegal*, *the ycf1* gene is present at the J_SA_ junction, while in *V*. *nilotica*, it was found 14 bp away from J_SA_ in the IRa region (Fig 5).

### Phylogenetic analyses: Confirmation of recent classification based on complete CP

**Fig 6:**
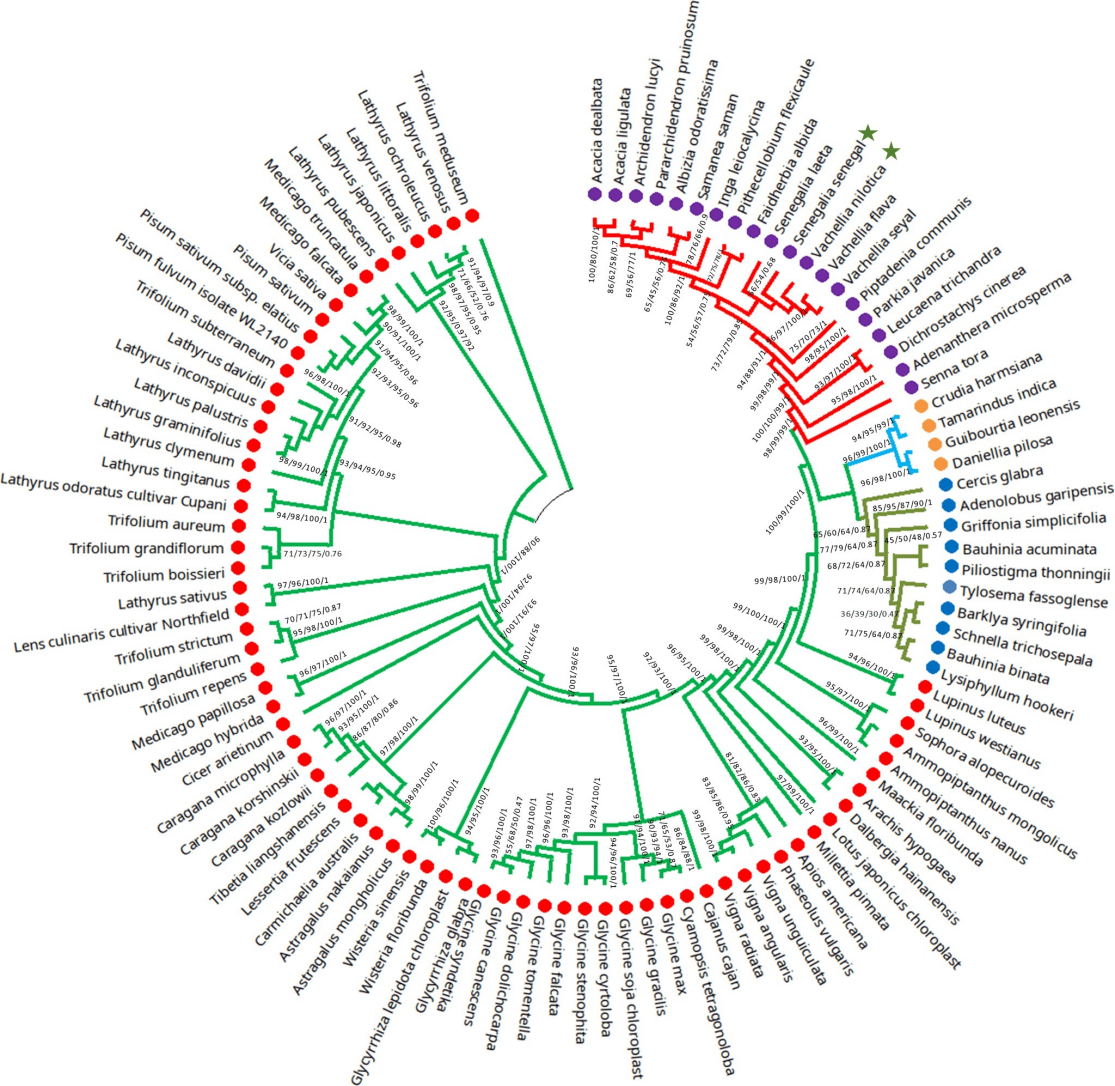
Phylogenetic tree constructed on the basis of whole genome dataset using four different methods: Bayesian inference (BI), maximum likelihood (ML), maximum parsimony (MP), and neighbor-joining (NJ). Numbers above the branches are the posterior probabilities of BI and bootstrap values for ML, MP and NJ. The star represents the position of *V*. *nilotica* and *S*. *senegal*.

Previously, numerous studies were conducted to resolve the phylogenetic position of Mimosoideae [46], but no study to date has investigated the basis of the complete chloroplast genome of *Vachellia* and *Senegalia* species. In this study, the phylogenetic position of *V*. *nilotica* and *S*. *senegal* within the family Fabaceae was established by analyzing multiple sequence alignments of complete cp genomes and 56 shared genes of 104 Fabaceae members (Fig 6 and S2). The 56 shared genes (from all species) and the complete cp genome sequence generated phylogenetic trees with identical topologies (Fig 6 and S2). In these phylogenetic trees, *S*. *senegal* formed a sister clade with *S*. *laeta*, while *V*. *nilotica* shared a sister clade with *V*. *flava* and *V*. *seyal* with high posterior probability and bootstrap support values using four different methods (Fig 6). Our results supported the recent classification of *V*. *nilotica* and *S*. *senegal* in the genera *Vachellia* and *Senegal*, respectively, and did not support the former placement of these species in the genus *Acacia*.

## Discussion

This study reports the complete chloroplast genomes of *S*. *Senegal* and *V*. *nilotica*, ranging from 162.7~165.3 kb in length. Both cp genomes exhibit a typical quadripartite conserved structure, as reported for other angiosperm genomes [44, 47]. Both *V*. *nilotica* and *S*. *Senegal* encode 135 and 132 genes, including 89 and 87 protein-coding genes, respectively. Similar differences in the protein coding genes were also observed, as in previously reported genomes [46]. The important genes present in these genomes were also similar to those of previously reported angiosperm cp genomes [44, 48]. The main reason for size variation among the chloroplast genomes is the contraction and expansion in the IR regions of the genome [49]. The size variation was observed (161,681 bp ~178,887 bp) to be in keeping with the previously reported angiosperm genomes [46]. Genome conservation was observed in both genera with some minor changes in IR/SSC regions, which reveal evidence of variation in the chloroplast genomes and also provides some information in the evolutionary context of chloroplast genomes [50]. Divergence hotspots among the species facilitate comparative genomics, species identification [51] and phylogenetic studies at different levels [52]. Comparative analysis of these genomes through mVISTA revealed that coding regions, such as *rps16*, *rpoC2*, *atpF*, *rpoC1*, *accD*, *clpP*, *petD*, *rpl16*, *ycf1*, *ycf2* and *ndhA*, were more divergent than the non-coding regions, which is similar to the findings obtained with previously reported cp genomes [46, 53]. The significance of these divergent regions can be further used as potential DNA markers for phylogenetic studies, population genetics studies and species identification studies [54]. Some of the protein coding genes present in the plastid genomes were found to have versatile roles in the resolution of phylogenetic relationships of complex plant taxa, such as *rpoA*, *psal*, *petB* and *rps19* in *Notopterygium* species [55] and *ycf1* in *Anemopaegma* species [56]. Moreover, in some other species, such as *Veroniceae*, the *petD-rpoA*, *ycf4-cemA*, and *rpl32-trnL* genes were used for the identification of the species. In our study, the PCG regions were more conserved and showed less sequence divergence than the intergenic spacer region, which had a higher degree of divergence among the compared species. Surprisingly, the IR regions in these compared cp genomes were less divergent compared to the LSC and SSC regions, which were also previously reported [53].

Repetitive sequences within the chloroplast genome play a crucial role in evolution, divergence studies and cp genome rearrangement. Moreover, microsatellite-like SSRs play an important role in molecular-level identification and in population genetics [57, 58]. The identification of repetitive sequences in the IGS provides useful information in various angiosperm species [58]. Among all the compared genomes, *Albizia odoratissima* had the lowest number of total repeats (104), and *Acacia ligulata* had the highest number of total repeats in cp genomes of subfamily *Mimosoideae* [46] and among other angiosperms [59]. The *Adenanthera microsperma* genome was found to contain the highest number of palindromic repeats (26), and the lowest number (16) was reported in *Pithecellobium flexicaule*. The highest number of forward repeats (36) was found in *Pararchidendron pruinosum*, while the lowest (24) was found in *A*. *microsperma*. The tandem repeats were highest (91) in *Dichrostachys cinerea*, and the lowest (52) was in *Albizia odoratissima*. Plastome size variation leads to the variation in tandem repeats [60] and dispersed repeats as previously reported by [57]. Earlier studies also showed that these repeats play an important role in structural variation [61]. The highest number of SSRs among these genomes was 333 in *Parachidendron pruinosum*, while the lowest number of SSRs was observed in *A*. *microsperma*. This result was consistent with the previously reported chloroplast genome of wild roses [62].

The phylogenetic relationship of the genus *Vachellia* and *Senegalia* belonging to the sub-family Mimosoideae (Fabaceae) was poorly resolved previously using only a few plastid markers [63–65]. Phylogenomic analysis based on the complete chloroplast genome can be widely used to resolve the complex relationship at the family level, as previously reported in *orchiaceae* [66], and *Bambusoideae* [67]. A detailed comprehensive study of the subfamily Mimosoideae was reported by Wang et al.[46], but there was no mention of the phylogenomic placement of *V*. *nilotica* and *S*. *senegal* into the genus *Vachellia* and *Senegalia*. The results of our study indicate that phylogenetic trees based on the complete genome dataset and 56 shared genes of *V*. *nilotica* and *S*. *senegal* contain the same phylogenetic signals and support the recent classification of *V*. *nilotica* and *S*. *senegal* in the genera *Vachellia* and *Senegal*, respectively (Fig 6). A complete phylogeny of Mimosoideae was constructed to resolve the evolutionary relationship of Mimosoideae with Fabaceae. Structural rearrangement in the chloroplast genome is an important phylogenetic signal and is used to define monophyletic lineages in plant groups [68].

## Conclusion

We sequenced the chloroplast genome of *V*. *nilotica* and *S*. *senegal*. Both genomes shared the same gene organization and overall genome structure, which were also found in related species. The quadripartite structure (LSC/SSC/IRA/IRB) of the genomes was compared for Mimosoideae species, and no significant variation was noted in these genomes, instead showing the closest similarity to these species. The phylogenetic relationships of these species, which were formerly classified in the genus *Acacia* and later placed in the genera *Vachellia* and *Senegalia*, were validated on the basis of the complete chloroplast genome. Furthermore, the phylogenetic analyses revealed that both *V*. *nilotica* and *S*. *senegal* formed monophyletic clades, while *V*. *nilotica* further shared sub-monophyletic clades with *V*. *flava* and *V*. *seyal*, while the *S*. *senegal* shared the same clade with *S*. *laeta*. These findings may help to elucidate the complex taxonomy of these genera and the studied species *V*. *nilotica* and *S*. *senegal*.

## Availability of data and materials

All data generated or analyzed during this study are included in this published article.

## Supporting information

S1 Fig Alignment visualization of the *V*. *nilotica* and *S*. *Senegal* chloroplast genome sequences. VISTA-based identity plot showing sequence identity among nineteen species, using *V*. *nilotica* as a reference genome. The vertical scale indicates the percentage of identity, ranging from 50% to 100%. The horizontal axis indicates the coordinates within the chloroplast genome. Arrows indicate the annotated genes and their transcriptional direction.
(PDF)

S2 Fig Phylogenetic trees of *V*. *nilotica* and *S*. *senegal* based on protein coding genes (PCGs). A phylogenetic tree was constructed for 104 species from the family Fabaceae based on 56 shared protein coding genes. The following four different methods were used for the 56 shared gene data sets: Bayesian inference (BI), maximum likelihood (ML), maximum parsimony (MP), and neighbor-joining (NJ). Numbers above the branches are the posterior probabilities of BI and bootstrap values for ML, MP and NJ.
(PDF)
