# Supplementary figures and images for: Complete Chloroplast Genomes of Vachellia nilotica and Senegalia senegal: Comparative Genomics and Phylogenomic Placement in a New Generic System
Source: PLoS One. 2019 Nov 25;14(11):e0225469. doi: 10.1371/journal.pone.0225469 (PMC6876885; doi:10.1371/journal.pone.0225469)

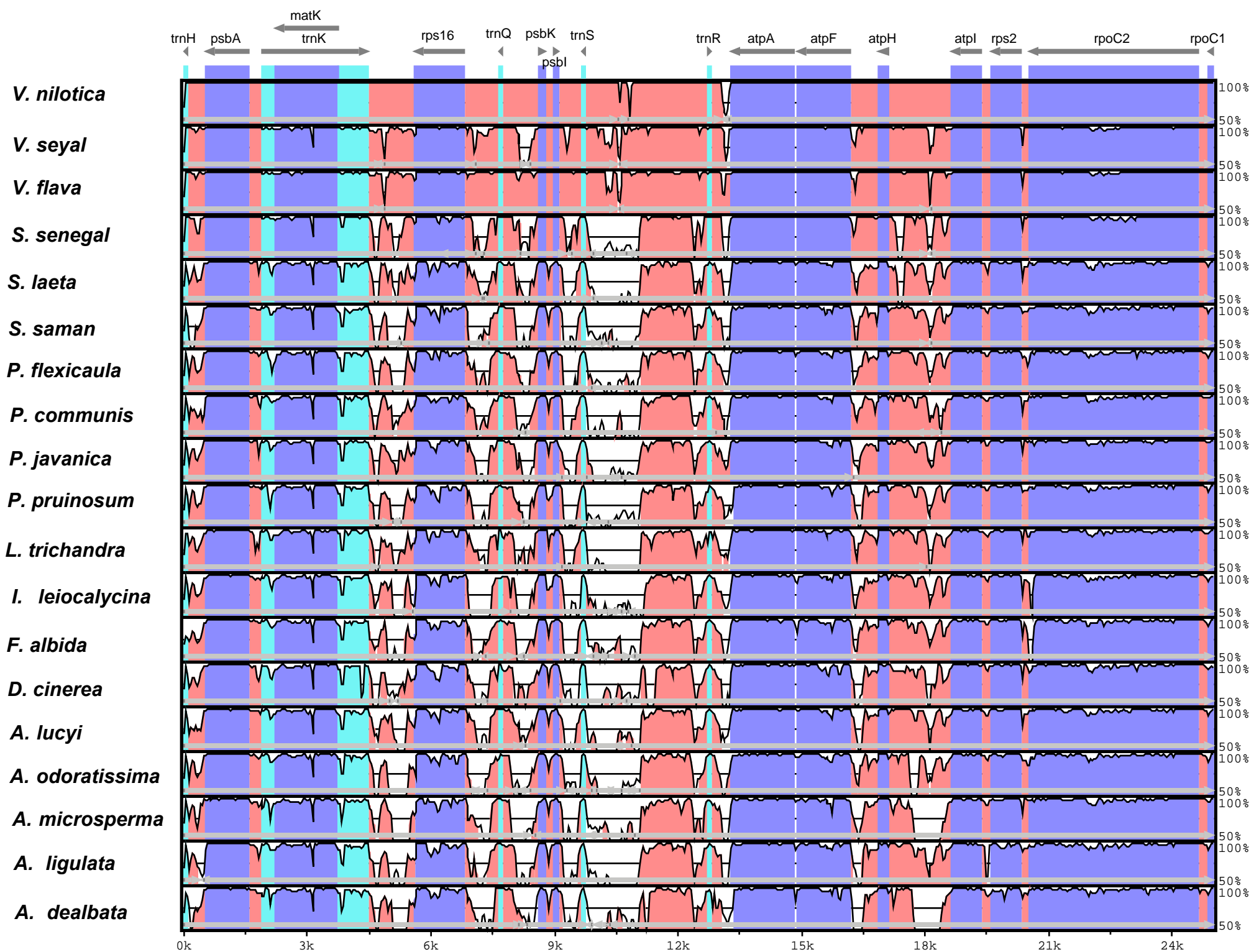

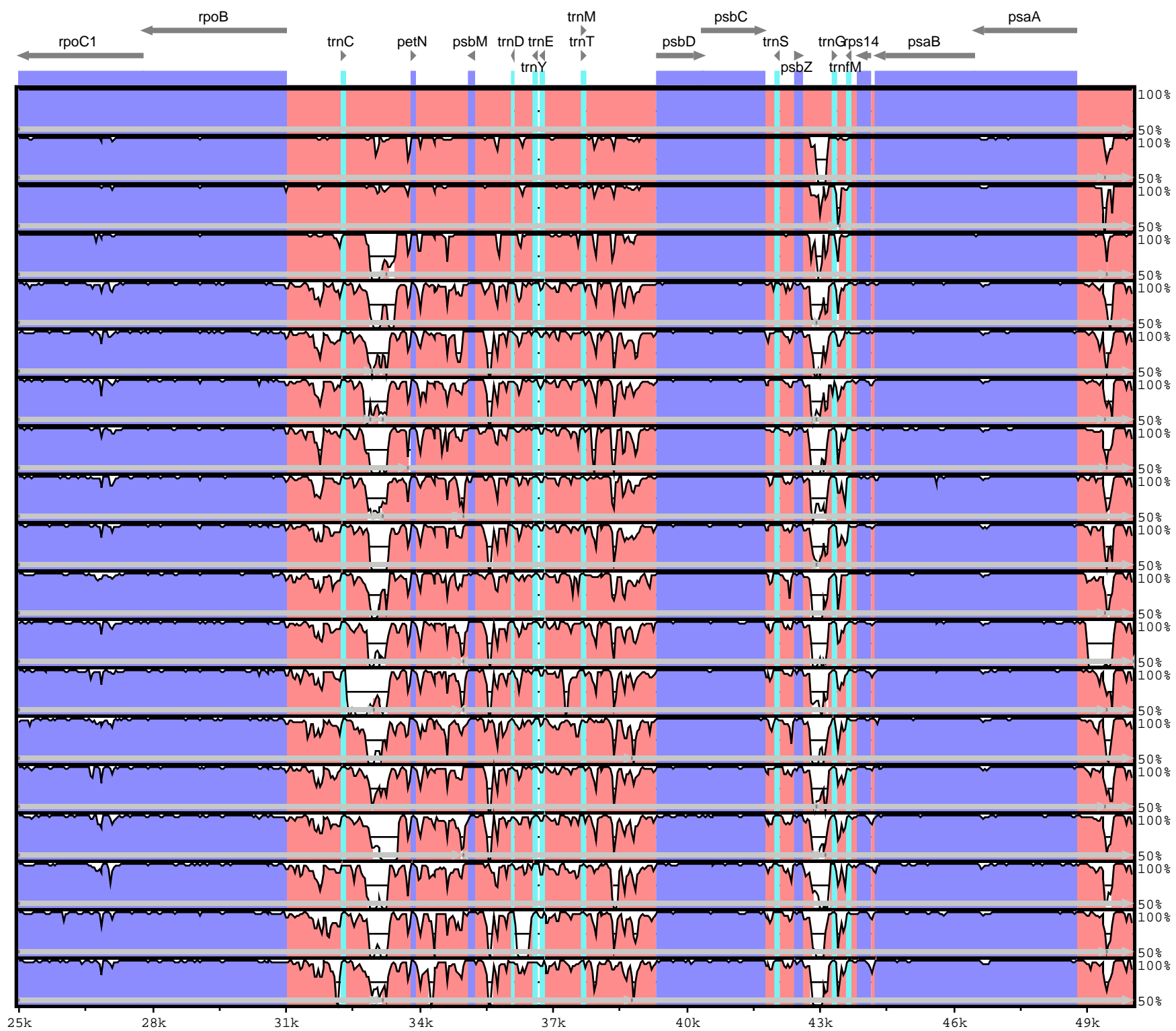

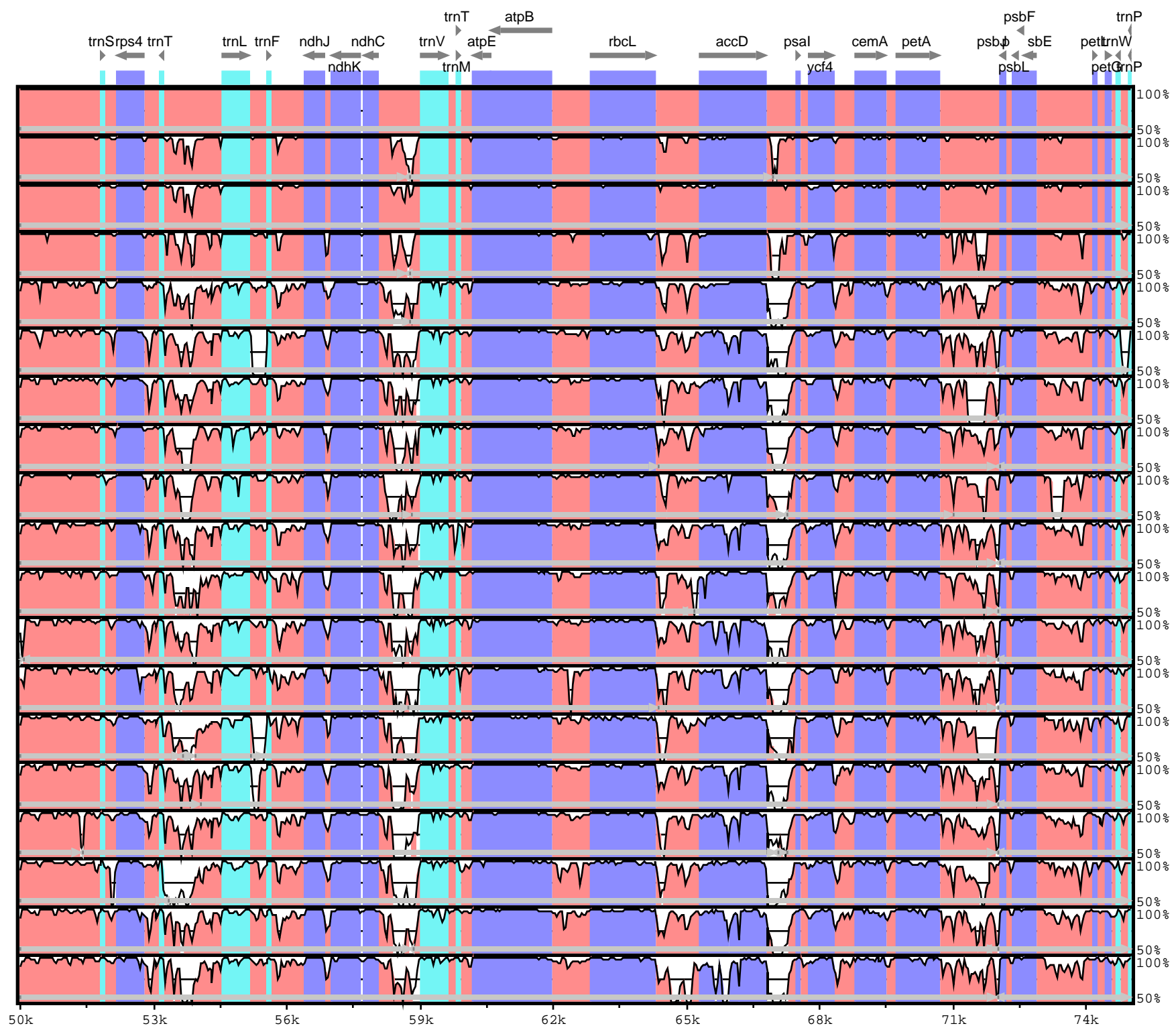

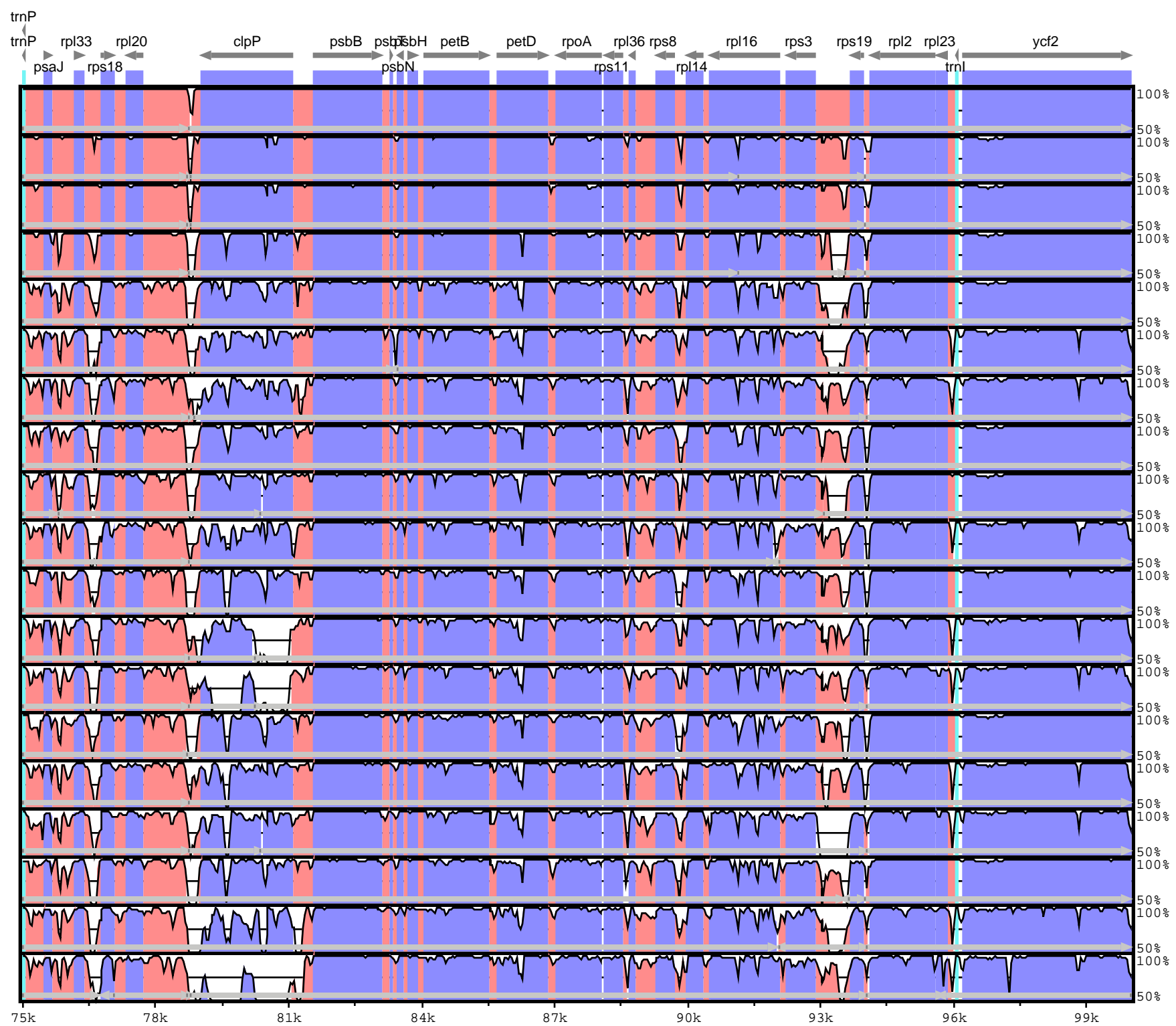

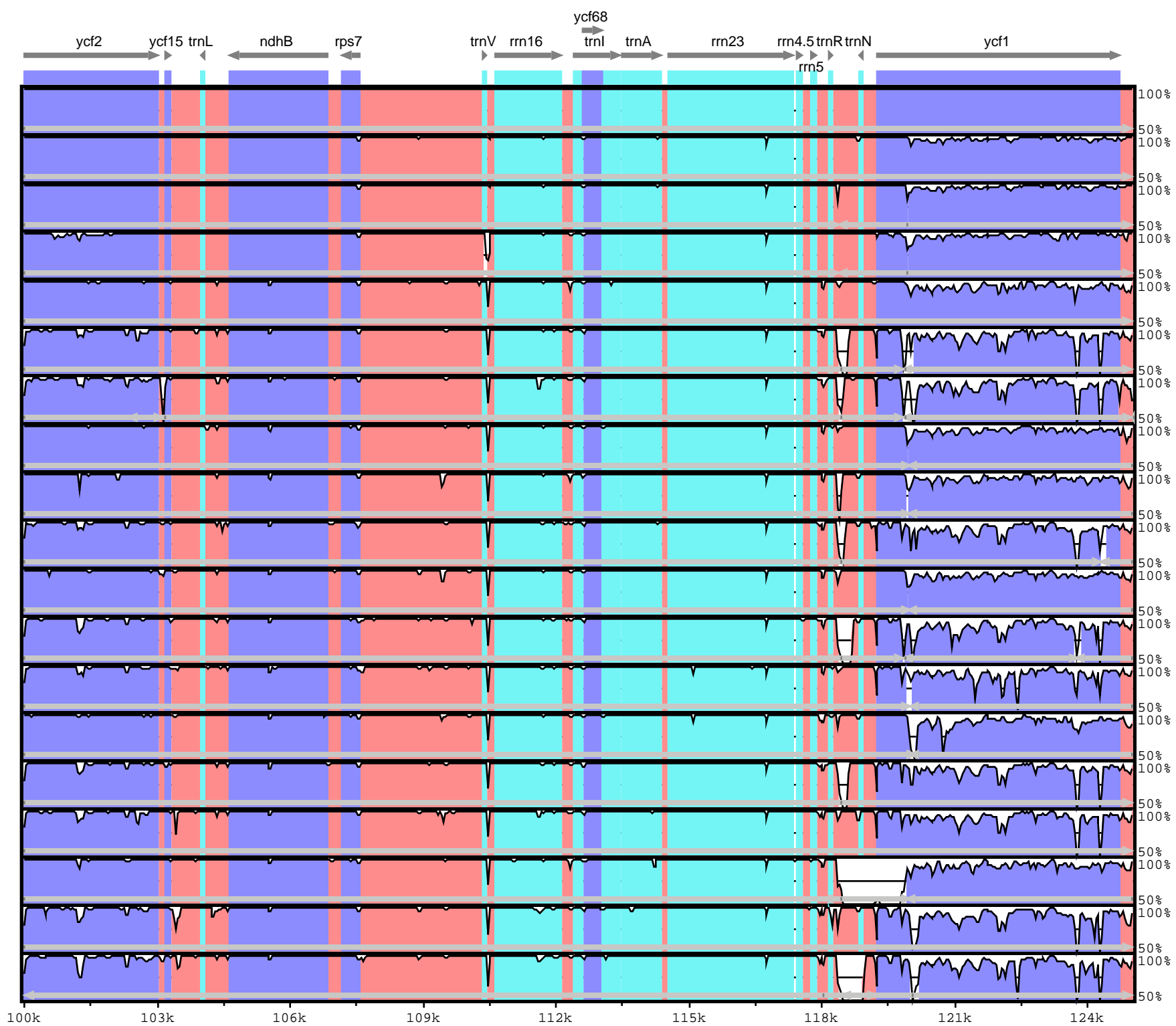

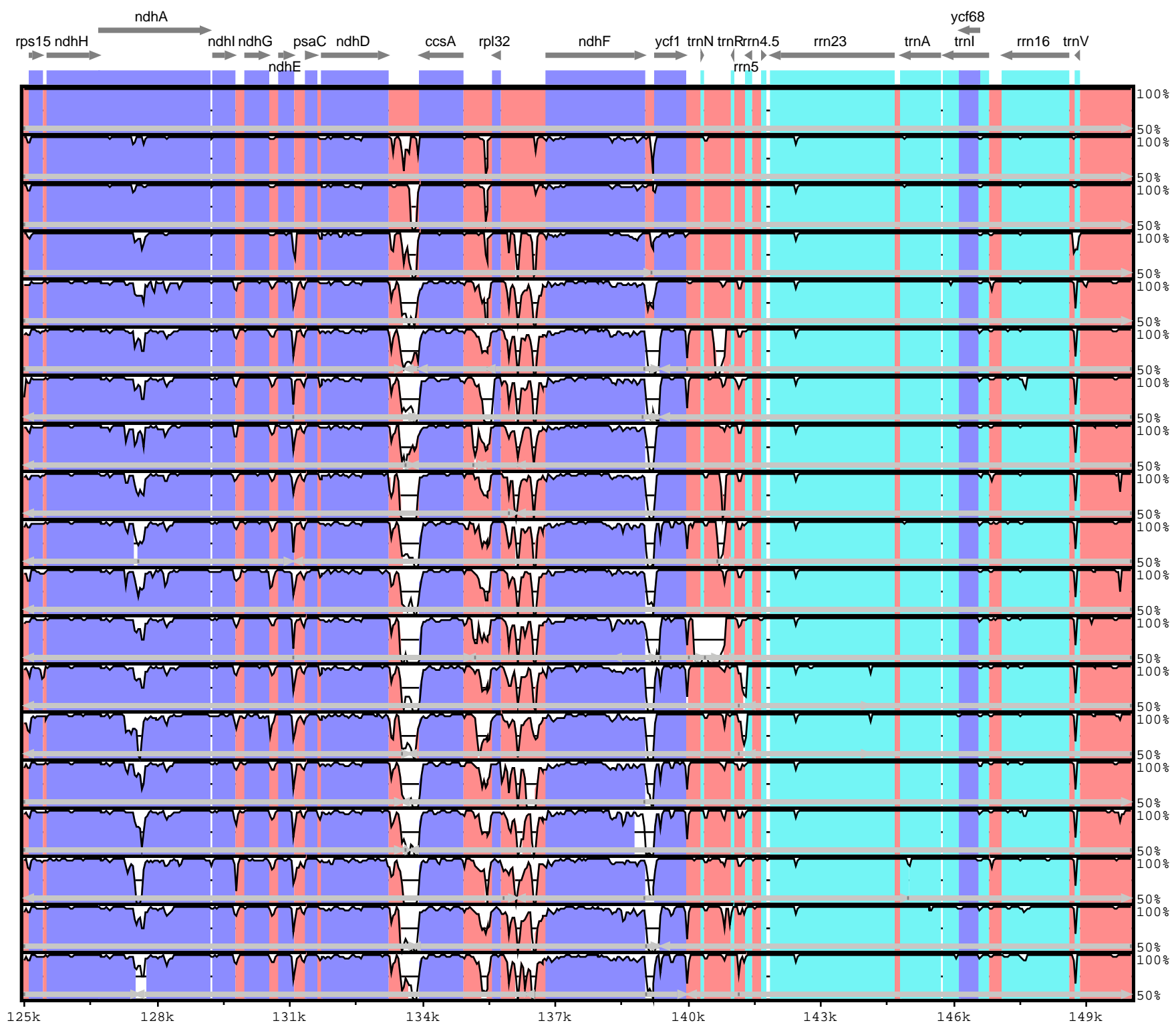

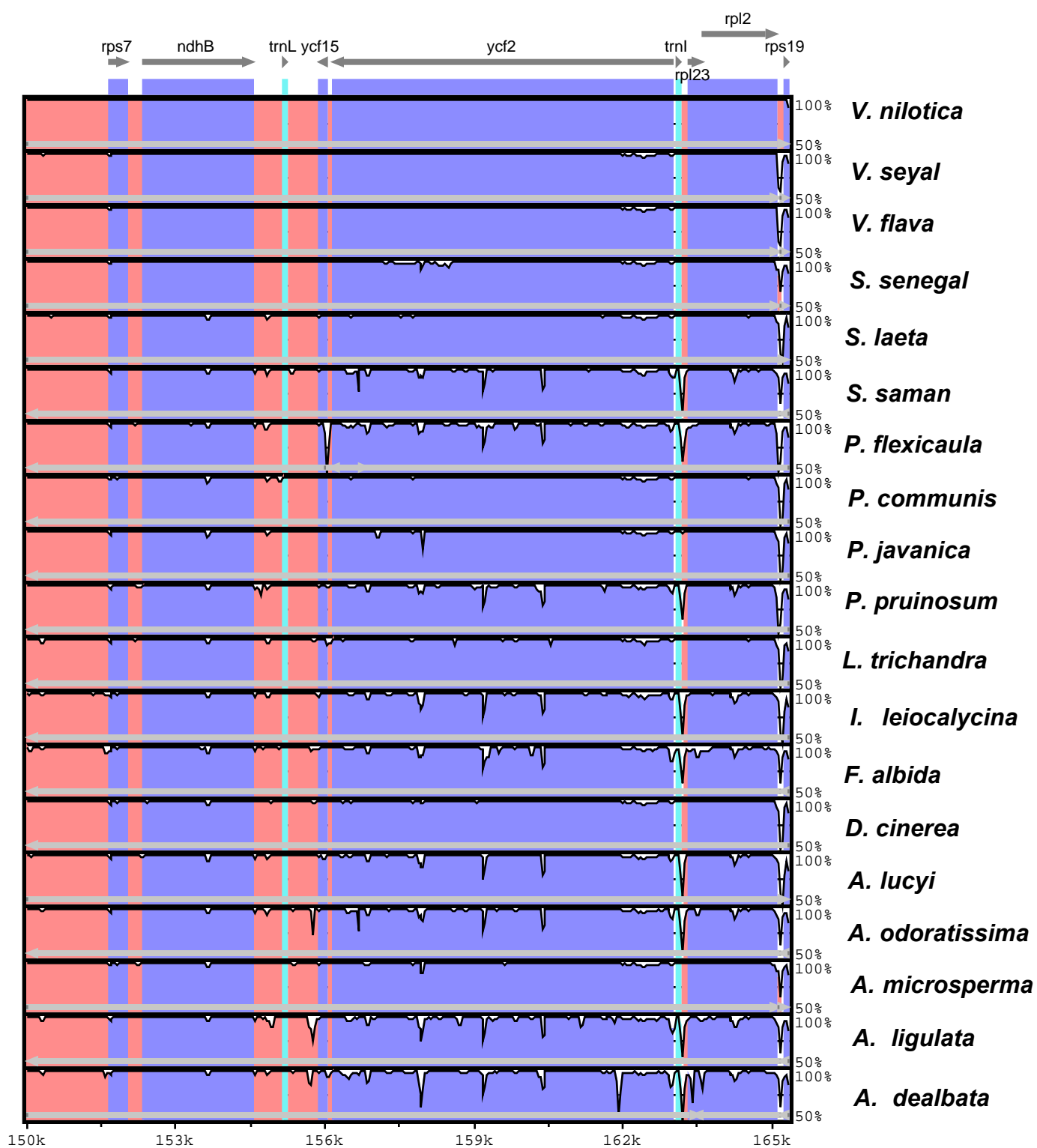

Supplement: S1 Fig — VISTA-based identity plot showing sequence identity among nineteen species, using V. nilotica as a reference genome. The vertical scale indicates the percentage of identity, ranging from 50% to 100%. The horizontal axis indicates the coordinates within the chloroplast genome. Arrows indicate the annotated genes and their transcriptional direction. (PDF) [file pone.0225469.s001.pdf]

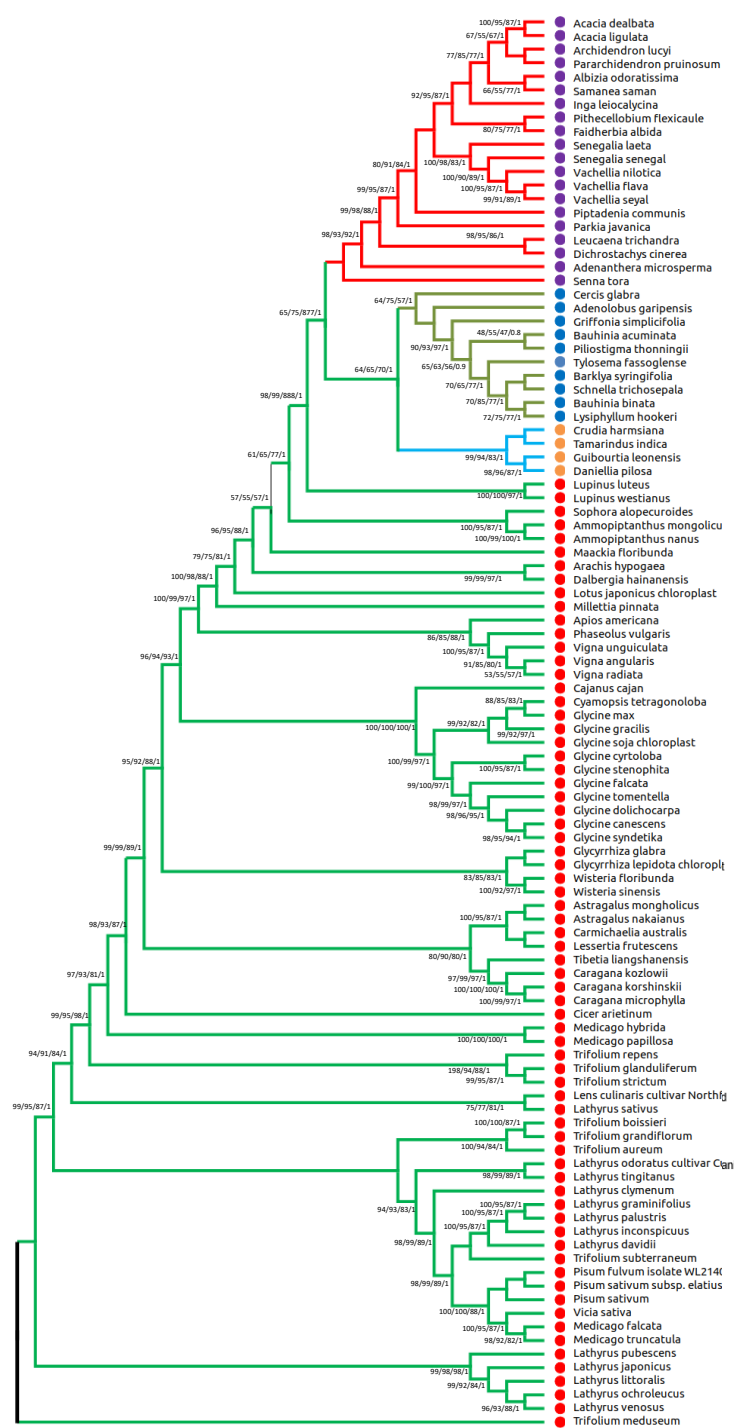

Caesalpinioideae

Cercidoideae

Detarioideae

Papilionoideae

Supplement: S2 Fig — A phylogenetic tree was constructed for 104 species from the family Fabaceae based on 56 shared protein coding genes. The following four different methods were used for the 56 shared gene data sets: Bayesian inference (BI), maximum likelihood (ML), maximum parsimony (MP), and neighbor-joining (NJ). Numbers above the branches are the posterior probabilities of BI and bootstrap values for ML, MP and NJ. (PDF) [file pone.0225469.s002.pdf]
